# Targeted gene disruption by use of transcription activator-like effector nuclease (TALEN) in the water flea *Daphnia pulex*

**DOI:** 10.1186/s12896-014-0095-7

**Published:** 2014-11-18

**Authors:** Chizue Hiruta, Yukiko Ogino, Tetsushi Sakuma, Kenji Toyota, Shinichi Miyagawa, Takashi Yamamoto, Taisen Iguchi

**Affiliations:** Okazaki Institute for Integrative Bioscience, National Institute for Basic Biology, National Institutes of Natural Sciences, Higashiyama 5-1, Myodaiji, Okazaki, Aichi 444-8787 Japan; Faculty of Life Science, Graduate University for Advanced Studies (SOKENDAI), 5-1 Higashiyama, Myodaiji, Okazaki, Aichi 444-8787 Japan; Department of Mathematical and Life Sciences, Graduate School of Science, Hiroshima University, Higashi-Hiroshima, 739-8526 Japan

**Keywords:** *Daphnia pulex*, *Distal*-*less*, Platinum TALEN, Gene disruption, Knock-out, Targeted mutagenesis, Gene manipulation, Genome editing

## Abstract

**Background:**

The cosmopolitan microcrustacean *Daphnia pulex* provides a model system for both human health research and monitoring ecosystem integrity. It is the first crustacean to have its complete genome sequenced, an unprecedented ca. 36% of which has no known homologs with any other species. Moreover, *D. pulex* is ideally suited for experimental manipulation because of its short reproductive cycle, large numbers of offspring, synchronization of oocyte maturation, and other life history characteristics. However, existing gene manipulation techniques are insufficient to accurately define gene functions. Although our previous investigations developed an RNA interference (RNAi) system in *D. pulex*, the possible time period of functional analysis was limited because the effectiveness of RNAi is transient. Thus, in this study, we developed a genome editing system for *D. pulex* by first microinjecting transcription activator-like effector nuclease (TALEN) mRNAs into early embryos and then evaluating TALEN activity and mutation phenotypes.

**Results:**

We assembled a TALEN construct specific to the *Distal*-*less* gene (*Dll*), which is a homeobox transcription factor essential for distal limb development in invertebrates and vertebrates, and evaluated its activity *in vitro* by single-strand annealing assay. Then, we injected TALEN mRNAs into eggs within 1 hour post-ovulation. Injected embryos presented with defects in the second antenna and altered appendage development, and indel mutations were detected in *Dll* loci, indicating that this technique successfully knocked out the target gene.

**Conclusions:**

We succeeded, for the first time in *D. pulex*, in targeted mutagenesis by use of Platinum TALENs. This genome editing technique makes it possible to conduct reverse genetic analysis in *D. pulex*, making this species an even more appropriate model organism for environmental, evolutionary, and developmental genomics.

**Electronic supplementary material:**

The online version of this article (doi:10.1186/s12896-014-0095-7) contains supplementary material, which is available to authorized users.

## Background

Environmental cues constantly influence gene expression; hence interactions among them are indispensable for animal adaptations against changing environments. The water flea *Daphnia pulex* shows a striking ability to contend with environmental changes, resulting in various adaptive phenotypes involving traits such as body size, longevity, behavior, and morphology [1]. It is well known that daphnids occupy a key position as the intermediate link between primary productivity and top predators in the aquatic food chain, and they also serve as an environmental indicator organism because of their high sensitivity to water quality [2]. These characteristics, in addition to their short life cycle, large brood sizes, and synchronization of oocyte maturation, make them valuable for environmental, evolutionary, and developmental genomics studies. Furthermore, the *D. pulex* genome has been sequenced [3], which facilitates the identification of candidate genes involved in their unique biological attributes. The analysis of transcriptomics, proteomics, and metabolomics also showed profiles of a large number of putative factors involved in their unique life history characteristics (cf. [4,5]). The *D. pulex* genome possesses as many as about 31,000 genes; this large suite of genes may provide the arsenal responsible for the organism’s responsiveness to environmental challenges [3]. So far, however, there are no effective methods for manipulating genes, and only transient analysis of gene function by the RNA interference (RNAi) method [6] is available. The RNAi system in *D. pulex* suffers from several weaknesses, including incomplete silencing, transient effects, and limited analyzable stages. Because of these situations, even in the post-genomic era, the establishment of a gene manipulation technique has been eagerly anticipated to address the characterization of gene function in *D. pulex*.

There are three main types of artificial sequence-specific nucleases based on the source mechanism of DNA binding that guides nuclease activity to a genomic target: zinc-finger nucleases (ZFNs), transcription activator-like effector nucleases (TALENs), and clustered regularly interspaced short palindromic repeats (CRISPR) [7]. In this study, we chose TALEN to edit the *D. pulex* genome, although each method has both advantages and disadvantages in terms of cost, sequence-specificity, off-target effects, and so on [8,9]. The transcription activator-like effectors (TALEs) were first discovered in the plant pathogen *Xanthomonas* sp., and are composed of a conserved central domain for site-specific DNA binding [10,11]. The DNA-binding domain consists of varying numbers of tandem repeats of a 34-amino acid monomer, which specifies the DNA-binding sequence by its 12th and 13th amino acids, called repeat-variable di-residues (RVDs). RVDs specifically recognize a single nucleotide with the following codes: NG = T, HD = C, NI = A, NN = G or A [10,12,13]. Sakuma et al. [14] demonstrated that TALENs with periodically-patterned repeat variants harboring non-RVD variations, called Platinum TALENs, showed higher activities than TALENs without non-RVD variations (so-called Golden TALENs). TALENs are artificially generated by fusing TAL effector DNA-binding domains to a Fok I nuclease domain, and successfully harnessed to custom-designed sequence-specific nucleases [15,16]. TALENs induce DNA double-strand breaks (DSBs) that can be repaired by the error-prone non-homologous end joining (NHEJ) system to cause insertion and/or deletion mutations at targeted genomic loci.

TALEN-mediated gene targeting has been applied in a great number of vertebrates and invertebrates [7,9,17]. In arthropods, it has been reported in insect species including flies [18], mosquitos [19,20], crickets [21], and silkworms [22]. Very recently, mutagenesis of an eye development gene was achieved in one congener *Daphnia magna* using the CRISPR/Cas system [23]. However, the conditions developed in *D. magna* are not directly applicable to *D. pulex*, possibly because of the difference in egg size and contents, and developmental duration, as described in our previous study [6].

*Distal*-*less* (*Dll*) and its homologs, *Dlx* genes, which function as homeodomain transcription factors, play one of the major roles in distal limb development throughout the animal kingdom [24]. Reduction of Dll activity caused defects of distal appendage segments in arthropods, resulting in the production of an easily recognizable phenotype (cf. [25,26]). In addition, the results of *Dll* RNAi in *D. pulex* [6] provide possible comparative data regarding phenotype, which is why this endogenous developmental gene was selected as a target for proof-of-principle TALEN in *D. pulex*.

The goal of this study was to develop a targeted gene disruption system by TALEN-mediated artificial DSBs in *D. pulex*. A TALEN target site was designed and assembled, and then TALEN mRNAs were microinjected into embryos to successfully induce insertion and/or deletion mutations.

## Results and discussion

### Construction and evaluation of *Dll* TALENs

**Figure 1:**
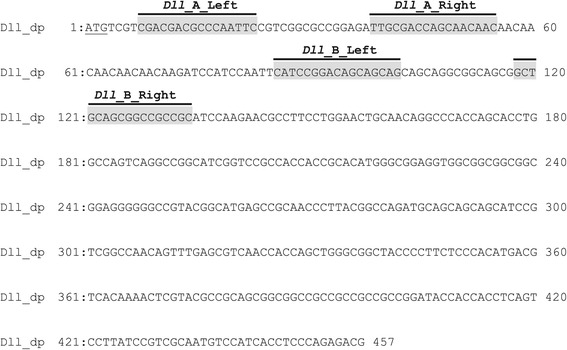
**The first exon nucleotide sequence of the** ***D*** **.** ***pulex Dll*** **gene.** Two pairs of TALENs, *Dll*_A and *Dll*_B, were designed in the first exon (highlighted in gray). The spacer regions of *Dll*_A and *Dll*_B each contain 15 bp nucleotides. Underline indicates ATG start codon.

**Figure 2:**
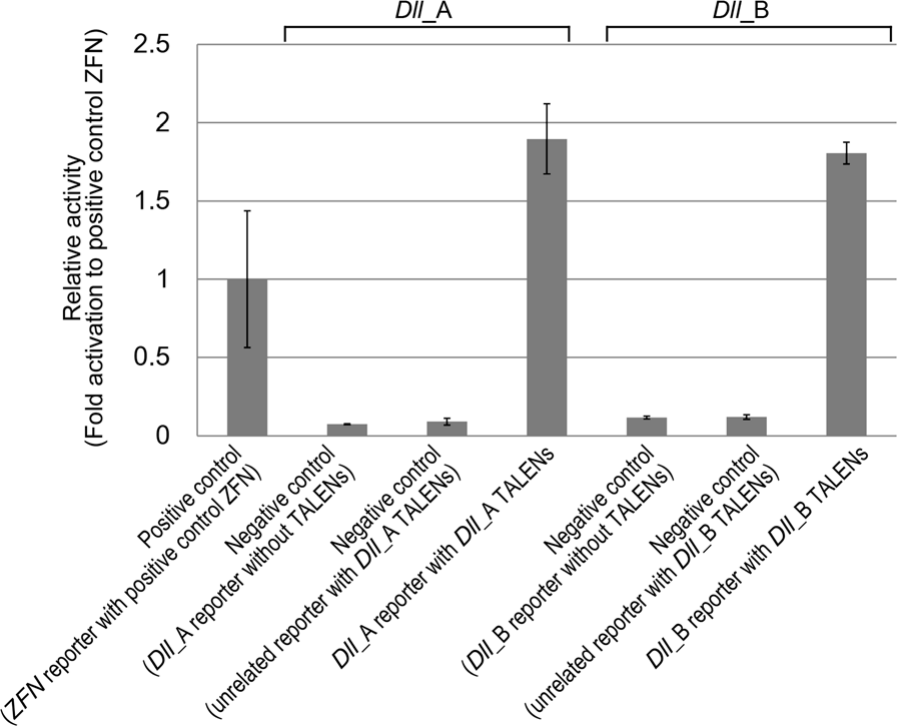
**Activity evaluation of** ***Dll*** **_A and** ***Dll*** **_B TALENs by SSA assay.** TALEN plasmids and the corresponding reporter plasmid, TALEN plasmids and unrelated reporter plasmid, or the reporter plasmid without TALEN plasmids were transfected into HEK293T cells. After the luciferase assay, fold activation scores against the positive control ZFN (pSTL-ZFA36 with the corresponding reporter) [28] were calculated. Data are expressed as means ± SEM (n = 3).

We first cloned and sequenced the partial sequence of the *Dll* gene in *D. pulex*, and then designed two pairs of TALENs, *Dll*_A and *Dll*_B, targeted to the first exon to induce a nonsense mutant protein (Figure 1). After constructing the TALENs by the Platinum Gate TALEN Kit, the activities of TALENs were evaluated by human cell-based single-strand annealing (SSA) assay [27] (Figure 2). Both *Dll*_A and *Dll*_B TALENs showed higher scores than the positive control ZFN [28], suggesting that both TALENs have DSB-inducing activities. We have evaluated the TALEN activities using human cells for various animal applications including sea urchin [29] and fruit fly [27,30], suggesting that the activity score measured in human cells is, in principle, a good indication of the level of inducing DSBs in a wide range of animals. SSA assay can directly be performed with the constructed TALEN plasmids in human cells, so that we can easily know the quality of the constructed TALENs before making mRNAs and injecting them into the animals. We thus think the validation process with human cell-based SSA assay is beneficial to apply TALENs in animals including *D. pulex*.

### Detection of mutations and their sequences

**Figure 3:**
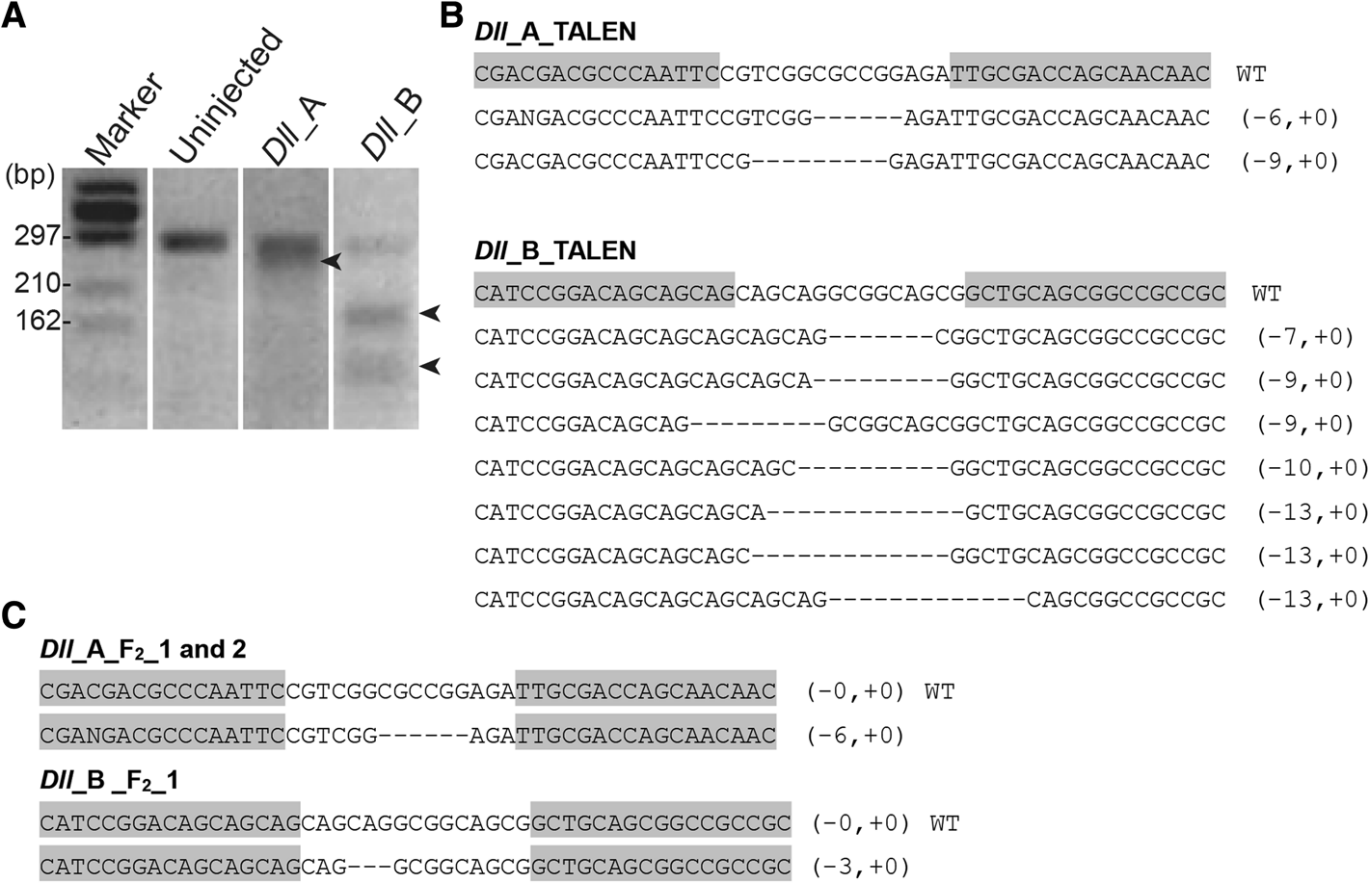
**Detection of TALEN-induced mutations and their sequences. (A)** Mutations in *Dll* were detected by T7EI assay. Arrowheads indicate the cleavage products generated in T7EI assay of F_0_ founders. **(B)** Sequence analysis of *Dll* mutant alleles in F_0_ founders induced by TALENs. The wild type (WT) sequence is shown at the top with the TALEN-binding sites (highlighted in gray). The number of nucleotides deleted (-) and inserted (+) is indicated in parentheses. **(C)** Sequence analysis of *Dll* mutant alleles in F_2_ embryos. Binding sites and insertions/deletions indicated as in **(B)**.

Two pairs of *Dll* TALEN mRNAs were microinjected into eggs just after ovulation, and the genome modification efficiencies *in vivo* were evaluated by a T7 endonuclease I (T7EI) assay. We found that a signal intensity of undigested band in the TALEN-injected embryos was weaker than that in uninjected embryos (Figure 3A). Furthermore, multiple digested bands were detected in *Dll*_B-injected embryos, whereas only a slightly separated band was detected in *Dll*_A-injected embryos. We thus concluded that *Dll*_B exhibits higher genome modification efficiencies rather than *Dll*_A.

To investigate the successful genome modifications of the F_0_ founder line, the genomic region surrounding the TALEN target was amplified and subcloned. Sequencing analysis revealed that TALENs predominantly induce small indel mutations ranging in size from 6-13 bp (Figure 3B), which is similar to results obtained in other arthropods [18-22]. In *Dll*_A-injected F_0_ founders, only two types of mutations, 6- and 9-nucleotide deletions, were detected. These mutations did not cause a frame shift and growth defect (see next section), suggesting that the gene function was not compromised. On the other hand, seven types of mutations were detected in *Dll*_B-injected F_0_ founders, five of which induced a frame shift leading to the appearance of a stop codon in the first exon. These mutations are predicted to result in a very short protein (83 amino acids) without a homeodomain, which is a functional DNA-binding domain and highly conserved among other arthropods. The variations of mutation patterns in *Dll*_B-injected F_0_ founders were higher than those in *Dll*_A-injected F_0_ founders, as expected from the T7EI assay. Based on these results, we conclude that the T7EI assay is a quick and easy method for estimating TALEN *in vivo* activity in F_0_ embryos of *D. pulex*.

To investigate whether these mutations were heritable, we then cloned and sequenced the target genomic region from F_2_ embryos. DNA sequencing revealed that inherited modifications existed in the two and one F_2_ lines injected with *Dll*_A and B, respectively (Figure 3C). Progeny from the same F_0_ founder had the same mutation genotype, indicating that the F_0_ founder carried a single germline mutation, not a mosaic germline mutation. Because cleavage proceeds without cytokinesis during injection period [31] and TALEN mRNAs can easily diffuse throughout the egg, it is considered that this method is able to induce mutation at very early developmental stages and easily establish mutant lines having a single germline mutation, without a need for complicated screening. In other words, mutation analysis can be carried out in a mere generation.

### Phenotype of *Dll* TALEN in *D. pulex*

Results from TALEN experiments are summarized in Table 1. Compared to uninjected embryos, the low viability of the embryos injected with *Dll* TALEN mRNAs may be partly attributed to damage due to microinjection and/or toxicity of TALEN mRNAs, as well as the embryonic lethality of *Dll* knock-out mutants. The difference of viability and/or morphological phenotype among embryos injected with *Dll*_A or *Dll*_B might depend on their genetic phenotype, such as non-mosaic/mosaic, monoallelic/biallelic, and non-frame shift/frame shift mutations. When a half concentration of *Dll*_B (250 ng/μl) was injected, viability of injected embryos increased by 7.5% and two individuals did not have any prominent altered phenotypes, suggesting dose dependency of the TALENs on viability and phenotypes.

**Table 1:** **Summary of TALEN results**

**Platinum TALENs**	**mRNAs concentration (ng/μl)**	**Injected embryos**	**Juveniles**	**Viability (%)**	**Shortened 2** ^**nd**^ **antennae**
***Dll*** **_A**	500	88	36	40.9	0
***Dll*** **_B**	500	206	63	30.6	63
	250	134	51	38.1	49
**Uninjected**	-	130	107	82.3	0

**Figure 4:**
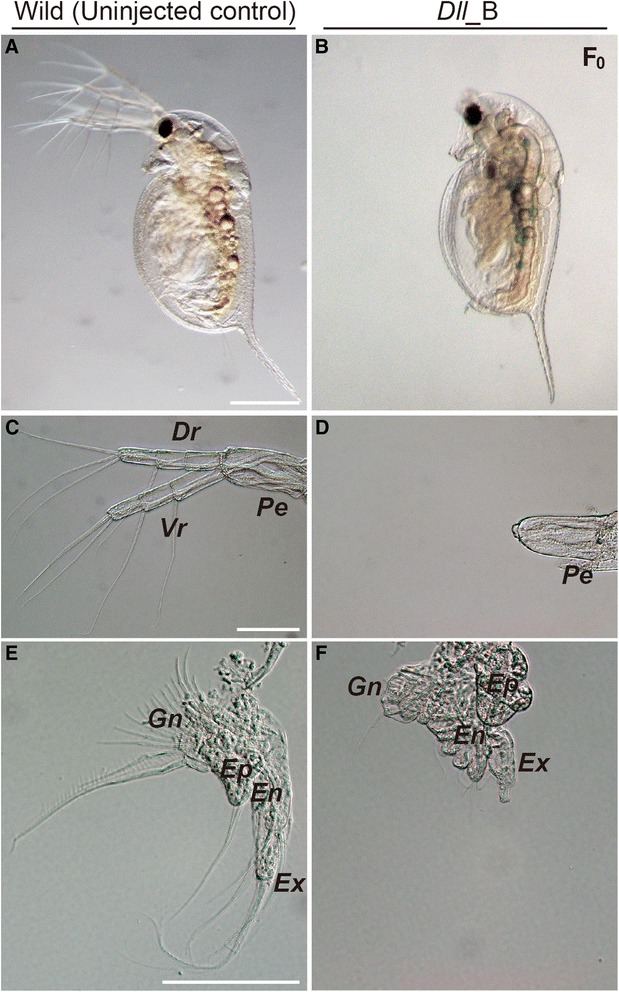
**Major** ***Dll*** **_B TALEN phenotypes.** The left and right columns show representative phenotypes of uninjected controls and individuals injected with *Dll*_B mRNAs, respectively. **(A, B)** Lateral view of first instar juvenile. **(C, D)** Lateral view of the second antennae. **(E, F)** Second thoracic limb (T2). *Dr*, dorsal ramus; *Ep*, epipodite; *En*, endopodite; *Ex*, exopodite; *Gn*, gnathobase; *Pe*, peduncle; *Vr*, ventral ramus. Scale bars =200 μm in **A**, **B**; 100 μm in **C**, **D** and **E**, **F**.

Morphologically, the *Dll*_A injected embryos did not show any prominent altered phenotypes, and both homozygous and heterozygous non-frame shift mutations were detected in *Dll* loci. Hence, we assessed the phenotype of *Dll*_B-injected F_0_ founders hereafter. Similar to the RNAi knockdown phenotype of *Dll* [6], *Dll*_B-injected F_0_ founders displayed various degrees of defects in second antennae, appendages, ocellus, abdominal claw, and abdominal setae, all tissues in which *Dll* is normally expressed (Figure 4 and Additional file 1: Figure S1). In the second antennae, the degree of the segment truncation was variably detected, ranging from severe (peduncle alone, lacking dorsal and ventral rami) to mild (peduncle and deficient rami). The first to fifth thoracic appendages, including each exopodite, were shortened. Moreover, a loss of the ocellus and abdominal setae, and minimized abdominal claw, were observed in *Dll*_B-injected embryos. We found that the mutants with severe defects in the second antenna and appendage development have biallelic mutations, failed to molt, and died before becoming adults. The lethality of *Dll* knock-out mutants was in accordance with previous studies on insects [25,32]. This easy and efficient induction of biallelic mutations by TALENs provides a valuable tool for parthenogenetic animals, including *Daphnia*, which are unable or poorly suited for conducting crossing experiments. In cases where monoallelic mutants are obtained, it is possible that the mutant line could be maintained and embryos reinjected with TALEN mRNAs to induce biallelic mutations.

Our results demonstrated that TALENs worked in *D. pulex* to induce heritable mutations into the endogenous genes. Moreover, TALENs can be used to induce more complicated mutagenesis other than micro-deletions or insertions. For example, Ma et al. [22] showed that simultaneous expression of two pairs of TALENs generates heritable large chromosomal deletions. Furthermore, Bedell et al. [33] and Zu et al. [34] demonstrated that co-injection of TALEN mRNAs and single-stranded oligodeoxyribonucleotides or donor vectors induced knock-in of small and large DNA fragments, respectively, into target loci via homologous recombination. All these technique based on TALENs will enable us to accurately define gene functions in *D. pulex* in future investigations.

## Conclusions

We developed a TALEN-mediated gene targeting model in *D. pulex*. Targeted mutagenesis can be attained in early embryos by injecting the right and left TALEN mRNAs into eggs within 1 hour post-ovulation. The genome editing by Platinum TALENs presented here, together with the full-genome sequence and other developed molecular tools, may significantly promote the genetic tractability of *D. pulex* as an important model for environmental, evolutionary, and developmental genomics.

## Methods

### *Daphnia* strain and culture conditions

The *D. pulex* strain [West Trenton (WTN6), collected in May 2006 by Sarah Schaak] was obtained from the Center for Genomics and Bioinformatics (Indiana University, USA). The strains were maintained in dechlorinated tap water, which was aerated and filtered through activated carbon for 2 weeks, at 18°C under artificial light conditions of 14 h light and 10 h dark to maintain reproduction. A 0.01-ml suspension of 10^9^ cells/ml *Chlorella vulgaris* was added every day to each culture (20-25 individuals/L). For rearing embryos, M4 culture medium (M4) was prepared using MilliQ water [35].

### Cloning of *Distal*-*less*

Total RNA was extracted using an ISOGEN kit (NIPPON GENE, Tokyo, Japan), and converted to cDNA using Superscript III and random primers (Life Technologies, Carlsbad, CA, USA) according to the manufacturer’s protocol. The *Dll* fragment was PCR amplified from the cDNA using a set of primers designed from the *Dll* sequence retrieved from wFleaBase http://wfleabase.org/ (Additional file 2: Table S1). Subsequently, the cDNA fragments were cloned into the pGEM-T Easy vector (Promega, Madison, WI, USA) according to the manufacturer’s instructions. Plasmids were sequenced using Sanger techniques that included the Big Dye terminator Ver. 3.1 (Life Technologies) on an ABI 3100 Avant or ABI 3130 Genetic Analyser DNA sequencer (Applied Biosystems Japan Ltd, Tokyo, Japan).

### TALEN target site design and assembly

The first exon sequences of *Dll* gene were scanned for potential TALEN target sites, which were identified using the TALEN Targeter program at https://tale-nt.cac.cornell.edu/node/add/talen. The following parameters were used: 1) spacer length: 12-16; 2) repeat array length of 15-20; 3) G substitute: NN; 4) Filter Options: Show all TALEN pairs (include redundant TALENs); 5) Streubel et al. guidelines: On. The Platinum Gate TALEN construction system described in Sakuma et al. [14] was used to assemble two pairs of TALENs, *Dll*_ A and *Dll*_ B (Figure 1), using the Platinum Gate TALEN Kit (Addgene, cat#1000000043). Assembled RVD repeats were as follows: HD NN NI HD NN NI HD NN HD HD HD NI NI NG NG HD for the left *Dll*_A TALEN, NN NG NG NN NG NG NN HD NG NN NN NG HD NN HD NI NI for the right *Dll*_A TALEN, HD NI NG HD HD NN NN NI HD NI NN HD NI NN HD NI NN for the left *Dll*_B TALEN, and NN HD NN NN HD NN NN HD HD NN HD NG NN HD NI NN HD for the right *Dll*_B TALEN. ptCMV-153/47-VR vectors (Addgene, included in the Platinum Gate TALEN Kit) were used as destination vectors.

### Single-strand annealing (SSA) assay in human cells

Construction of the reporter plasmids and the SSA assay was performed as previously reported [27]. Briefly, the oligonucleotides listed in Additional file 3: Table S2 were annealed and inserted into pGL4-SSA vector (Addgene, Plasmid 42962) to construct the reporter plasmids. The TALEN plasmids and/or reporter plasmids were transfected into HEK293T cells using Lipofectamine LTX (Life Technologies) according to the manufacturer’s instruction. At 24 hours post-transfection, a luciferase assay was performed using Dual-Glo Luciferase Assay System (Promega) and intensity of luminescence was measured with TriStar LB 941 plate reader (Berthold Technologies, Bad Wildbad, Germany).

### Injection of TALEN mRNAs into embryos

TALEN plasmid templates were linearized with Sma I (TaKaRa Bio, Shiga, Japan) and then transcribed *in vitro* using the mMESSAGE mMACHINE T7 Ultra kit (Ambion, Austin, TX, USA) according to the manufacturer’s protocol. The resultant mRNAs were purified using a NucAway Spin Columns (Ambion) and extracted with water-saturated phenol/chloroform, and finally resuspended in RNase-free water. Equal amounts of the right and left TALEN mRNAs were mixed to a final concentration of 500 and 250 ng/μl for microinjection and stored at –80°C until use. TALEN mRNAs were injected into the embryos as described previously [6].

### T7 endonuclease I (T7EI) assay

T7EI is a mismatch-resolving enzyme that can recognize heteroduplex DNAs and cleave DNA at single base pair mismatches. Thus TALEN-induced indel mutations can be detected. A pool of thirteen embryos collected at 48 h following injection with *Dll*_ A or *Dll*_B was used. Genomic DNA was isolated from TALEN-injected and uninjected embryos using DNeasy Blood and Tissue kit (Qiagen, Hilden, Germany). Additional file 2: Table S1 shows primers used to amplify the region containing the *Dll* target site from genomic DNA for T7EI assay and mutation analysis. PCR was performed using TaKaRa Ex Taq (TaKaRa Bio). Amplification conditions were: 98°C for 10 s, 56°C for 30 s, 72°C for 20 s, 45 cycles. T7 endonuclease I (New England Biolabs, Beverly, MA, USA) was added to PCR fragments and incubated at 37°C for 60 min. The samples were electrophoresed using 2% agarose gels.

### Mutation analysis

To confirm the presence of TALEN-mediated mutations, genomic DNA of each individual was isolated separately from twelve F_0_ embryos and nine F_2_ embryos injected with *Dll* _A or *Dll* _B as described above. The target genomic region was amplified with TaKaRa Ex Taq (TaKaRa Bio). Amplification conditions were: 98°C for 1 min; 35 cycles of 98°C for 10 s, 56°C for 30 s, 72°C for 20 s; 72°C for 1 min. The resulting fragments were subcloned into the pGEM-T Easy vector (Promega) and then sequenced as described above. For observation of morphological changes, first instar animals were examined under a stereomicroscope (M165 FC; Leica Microsystems GmbH, Wetzlar, Germany), and then fixed in ethanol and dissected with a pair of needles. The specimens were mounted in glycerin, observed by Nomarski differential interference contrast microscopy (Axioplan 2; Zeiss, Oberkochen, Germany), and recorded by digital camera (DP72; Olympus). The terminology used herein for morphology follows the usage of Stachowitsch [36].

## Additional files

Additional file 1: Figure S1. Phenotypes of *Dll*_B mRNAs injected juveniles. The left and right columns show representative phenotypes of uninjected controls and individuals injected with *Dll*_B mRNAs, respectively. **(A**, **B)** First thoracic limb (T1). The exopodite and endopodite were shortened by *Dll*_B TALEN mRNAs. **(C**, **D)** Third and fourth thoracic limbs (T3/4), having the same morphology. The exopodite was shrunk in *Dll*_B-injected juveniles. **(E**, **F)** Fifth thoracic limb (T5). The exopodite was shortened by *Dll*_B mRNAs. **(G**, **H)** Lateral view of the rostrum and head; an arrowhead indicates an ocellus. **(I**, **J)** Lateral view of abdomen; an arrow and arrowhead show an abdominal claw and abdominal seta, respectively. *Ep*, epipodite; *En*, endopodite; *Ex*, exopodite; *Fc*, filter comb. Scale bars =100 μm. Additional file 2: Table S1. The list of primer sequences. Additional file 3: Table S2. Oligonucleotides for construction of reporter plasmids.
