# Supplementary material for: Targeted gene disruption by use of transcription activator-like effector nuclease (TALEN) in the water flea Daphnia pulex
Source: BMC Biotechnol. 2014 Nov 18;14:95. doi: 10.1186/s12896-014-0095-7 (PMC4239399; doi:10.1186/s12896-014-0095-7)

**Additional file 1: Figure S1 Phenotypes of *Dll*_B mRNAs injected juveniles.**

The left and right columns show representative phenotypes of uninjected controls and individuals injected with *Dll*_B mRNAs, respectively. (A, B) First thoracic limb (T1). The exopodite and endopodite were shortened by *Dll*_B TALEN mRNAs. (C, D) Third and fourth thoracic limbs (T3/4), having the same morphology. The exopodite was shrunk in *Dll*_B-injected juveniles. (E, F) Fifth thoracic limb (T5). The exopodite was shortened by *Dll*_B mRNAs. (G, H) Lateral view of the rostrum and head; an arrowhead indicates an ocellus. (I, J) Lateral view of abdomen; an arrow and arrowhead show an abdominal claw and abdominal seta, respectively. *Ep*, epipodite; *En*, endopodite; *Ex*, exopodite; *Fc*, filter comb. Scale bars = 100 μm.


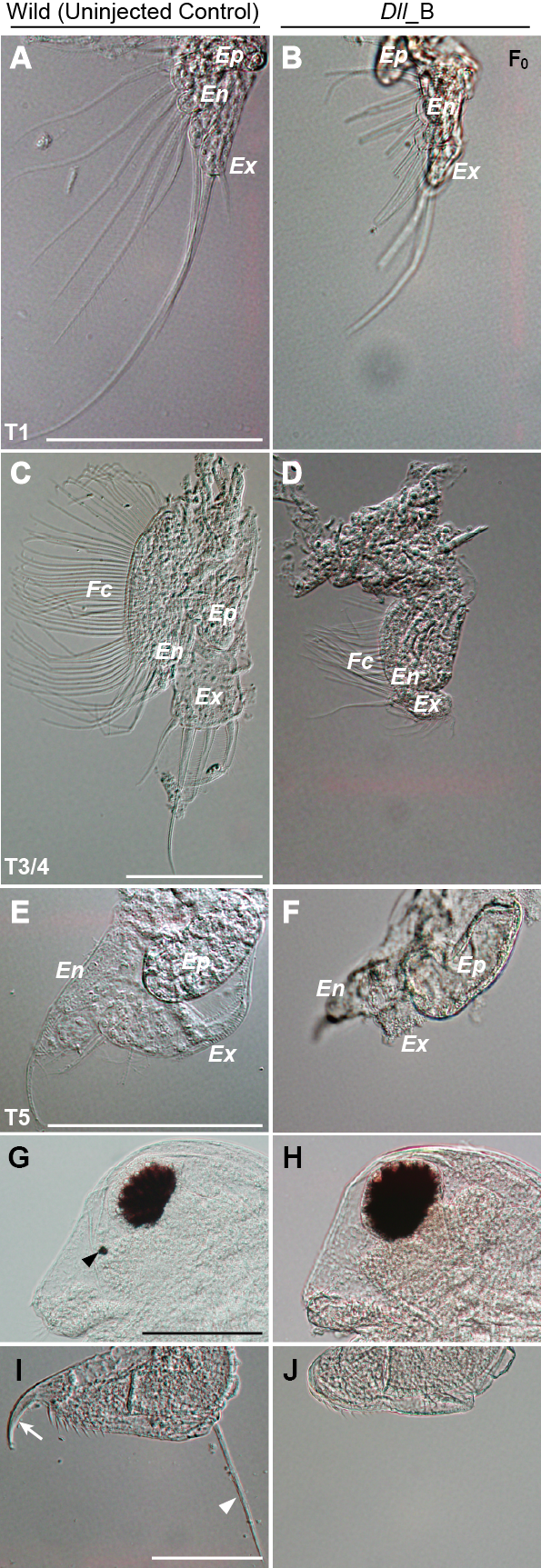

Supplement: Additional file 1: Figure S1. — Phenotypes of Dll_B mRNAs injected juveniles. The left and right columns show representative phenotypes of uninjected controls and individuals injected with Dll_B mRNAs, respectively. (A, B) First thoracic limb (T1). The exopodite and endopodite were shortened by Dll_B TALEN mRNAs. (C, D) Third and fourth thoracic limbs (T3/4), having the same morphology. The exopodite was shrunk in Dll_B-injected juveniles. (E, F) Fifth thoracic limb (T5). The exopodite was shortened by Dll_B mRNAs. (G, H) Lateral view of the rostrum and head; an arrowhead indicates an ocellus. (I, J) Lateral view of abdomen; an arrow and arrowhead show an abdominal claw and abdominal seta, respectively. Ep, epipodite; En, endopodite; Ex, exopodite; Fc, filter comb. Scale bars =100 μm. [file 12896_2014_95_MOESM1_ESM.docx]
