# Supplementary material for: Targeted gene disruption by use of transcription activator-like effector nuclease (TALEN) in the water flea Daphnia pulex
Source: BMC Biotechnol. 2014 Nov 18;14:95. doi: 10.1186/s12896-014-0095-7 (PMC4239399; doi:10.1186/s12896-014-0095-7)
Supplement: Additional file 2: Table S1. — The list of primer sequences. [file 12896_2014_95_MOESM2_ESM.docx]

**Additional file 2: Table S1 The list of primer sequences.**

| Application | Fwd (5’ to 3’) | Rev (5’ to 3’) |
| --- | --- | --- |
| Cloning | ATGTCGTCGACGACGCCCAA | AGATCCTCCTTCCGATTCGG |
| T7EI assay | ATGTCGTCGACGACGCCC | ACGAGTTTTGTGACGTCATGTG |
| Mutation analysis | ATGTCGTCGACGACGCCC | CATCTGGCCGTAAGGGTTG |
