# Supplementary material for: Targeted gene disruption by use of transcription activator-like effector nuclease (TALEN) in the water flea Daphnia pulex
Source: BMC Biotechnol. 2014 Nov 18;14:95. doi: 10.1186/s12896-014-0095-7 (PMC4239399; doi:10.1186/s12896-014-0095-7)
Supplement: Additional file 3: Table S2. — Oligonucleotides for construction of reporter plasmids. [file 12896_2014_95_MOESM3_ESM.docx]

**Additional file 3: Table S2 Oligonucleotides for construction of reporter plasmids.**

| Target site | Sense (5’ to 3’) | Antisense (5’ to 3’) |
| --- | --- | --- |
| *Dll*_A | GTCGGATCGACGACGCCCAATTCCGTCGGCGCCGGAGATTGCGACCAGCAACAACAGGT | CGGTACCTGTTGTTGCTGGTCGCAATCTCCGGCGCCGACGGAATTGGGCGTCGTCGATC |
| *Dll*_B | GTCGGATCATCCGGACAGCAGCAGCAGCAGGCGGCAGCGGCTGCAGCGGCCGCCGCAGGT | CGGTACCTGCGGCGGCCGCTGCAGCCGCTGCCGCCTGCTGCTGCTGCTGTCCGGATGATC |
